# Novel cell engineering of the Unfolded Protein Response to achieve efficient therapeutic protein production cell line

**DOI:** 10.1186/1753-6561-9-S9-P14

**Published:** 2015-12-14

**Authors:** Michael C Song, Jeff J Hou, Lars K Nielsen, Peter P Gray

**Affiliations:** 1 Australian Institute for Bioengineering and Nanotechnology (AIBN), The University of Queensland, St. Lucia, QLD, 4072, Australia

## Background

The bioproduction of therapeutic proteins such as monoclonal antibodies (mAb) continues to be a fast growing sector of advanced manufacturing. The ever increasing repertoire of therapeutic proteins coupled with the emergence of biosimilars led to increasing global demand for higher, faster, more cost-effective manufacturing process. Central to any good production platform is the capacity of the production cell line. A suitable production cell line exhibit physiological traits such as high specific productivity (qp), rapid doubling time, high peak cell density and efficient metabolism. The emergence of a suitable production cell with all the aforementioned traits is an extremely rare event, as it requires all facets of cellular transcription, translation, secretion and metabolic efficiency are individually optimized and collectively synchronized into a system capable of high level protein expression. One of the major cellular bottlenecks thought to limit protein production in mammalian cells lies in the cellular translation and secretion capacity. The over-expression of complex recombinant proteins such as mAbs driven by strong viral promoters exert considerable burden in the Endoplasmic Reticulum (ER) and Golgi apparatus. The increase in ER stress triggers the Unfolded Protein Response (UPR) which can result in cell apoptosis and possibly eliminating cells with high uptake of the Gene of Interest (GOI). We adopted host cell engineering approach using XBP1 spice ratio to expand cellular translation and secretion capacity to increase qp and improve the probability of isolating suitable high producers.

## Materials and methods

We screened a panel of mAb producing clonal cell lines using qPCR to identify XBP1 mRNA splice ratio as a suitable target to augment host cell translation and secretion machinery. We then employed FACS to isolate cell population with high ratio of spliced over unspliced XBP1 in order to overcome the negative regulatory effect of unspliced XBP1. We created a host cell line with high XBP1 splice ratio, exhibiting increased expression of chaperones, secretary vesicle proteins and quality assurance proteins without increasing UPR associated apoptotic markers as identified using qPCR. This host cell line was then used for in comparative mAb transient bioproduction studies with two different mAbs. Transient studies were performed using PEI-mediated transfection in industrial relevant chemically defined medium and feeding regime.

## Results

**Figure 1:**
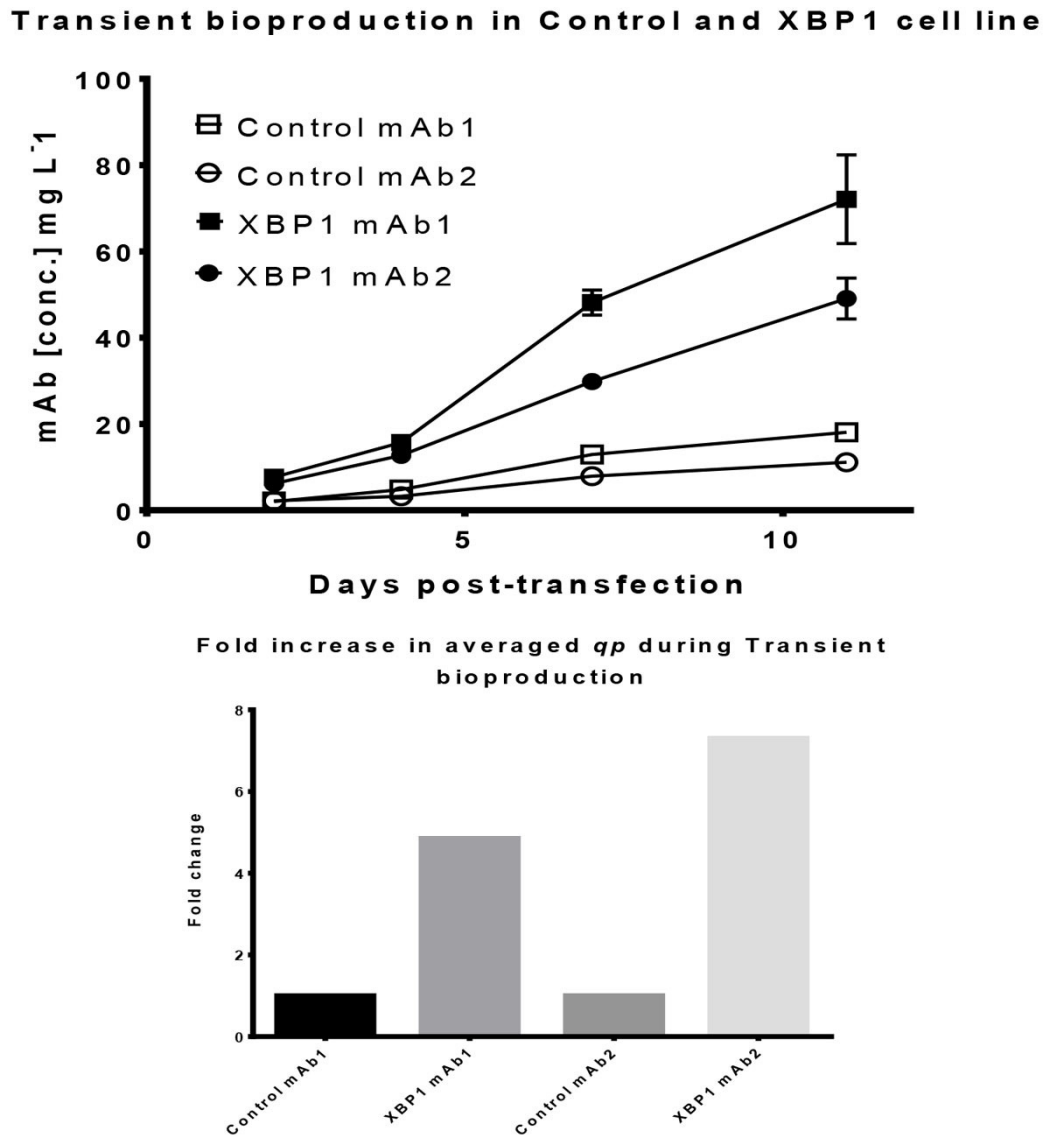
**Comparative transient bioproduction of mAbs between the Control CHO-K1 derived cell line and Translation and Secretion engineered XBP1 cell line**.

The XBP1 cell line demonstrated 7.5 fold increase in qp over the control cell line and more than 4 fold increase in final volumetric productivity (Figure 1). Moreover, we also observed 3-fold increase in the percentage of high producers with the XBP1 host cell line when we investigated the producer cell population distributions post-transfection. The XBP1 host cell derived mAb producing cells also demonstrated competency in conventional Stirred-tank bioreactor, showing promises of a commercially viable production platform.

## Conclusion

In conclusion, XBP1 splice ratio engineered host cell have expanded translation and secretion capacity to alleviate the ER stress experienced from during mAb bioproduction. The augmented capacity allowed for multi-fold improvements in qp and volumetric productivity and have the potential to increase the probability for isolating high producers. The engineered host cell line showed great promise to become a commercially suitable production platform cell line to significantly reduce the time and resources associated with cell line development.

